# Supplementary material for: Clients’ and genetic counselors’ perceptions of empathy in Japan: A pilot study of simulated consultations of genetic counseling
Source: PLoS One. 2023 Jul 19;18(7):e0288881. doi: 10.1371/journal.pone.0288881 (PMC10355444; doi:10.1371/journal.pone.0288881)
Supplement: Attachment — Submitted filename: Response_to_Reviewers.doc [file pone.0288881.s001.doc]

**Reviewer #1**:

Q1. The English should be reviewed by a native speaker for clarity and meaning.

A1. Thank you for your comment. The paper has been edited and rewritten by an experienced scientific editor again, who has improved the grammar and stylistic expression of the manuscript.

**Abstract**:

Q2. Clarity/meaning: “Client-counselor interactions about empathy involve a significant element in genetic counseling, which is an area of increasing need with the rapid acceleration of genetic testing across medical conditions and needs.”

A2. This sentence has been corrected to “Client-counselor interactions about empathy involve a significant element in genetic counseling, which is an area of increasing need with the rapid acceleration of genetic testing.”

Q3. Suggest changing: “This study aims to reveal the perceptions of empathy in clients and genetic counselors during simulated genetic counseling sessions” to “This study aimed to explore the perceptions of empathy in simulated genetic counseling consultations from the perspectives of clients and genetic counselors.”

A3. As indicated, the sentence has been changed. The term “consultations” has been used instead of “sessions” to refer to Reviewer 2’s comment. Kindly refer to Reviewer 2’s comments for your reference.

Q4. Suggest changing: “Semi-structured interviews with participants of simulated genetic counseling sessions and interpersonal process recall were used to elicit their experiences of empathy” to: “Semi-structured interviews and interpersonal process recall were used with participants of simulated genetic counseling consultations to elicit their experiences of empathy.”

A4. As indicated, the sentence has been changed.

Q5. Suggest changing: “A constructing grounded theory was used for data analysis” to: “A constructivist grounded theory was used for data analysis.”

A5. As indicated, the wording has been changed.

Q6. Suggest changing: “A total of 15 participants, 10 acting as clients and 5 as genetic counselors, participated in 10 simulated counseling sessions” to: “A total of 15 participants, including 10 clients and 5 genetic counselors, participated in 10 simulated counseling consultations.”

A6. As indicated, the sentence has been changed.

Q7. Clarity/meaning: “Clients’ perception represented genetic counselors’ empathic approaches, their subjective thoughts brought by the approaches and those changes”

Then suggest putting the sentence about counsellors attempts to demonstrate empathy first, and follow it with the sentence about clients reactions to the demonstrations of empathy.

A7. Thank you for your valuable suggestion. As per your recommendation, these sentences have been changed to “The genetic counselors attempted to demonstrate empathy and were sensitive toward detecting changes in clients. Meanwhile, the clients’ perceptions represented their feelings and thoughts elicited through the counselors’ empathic approaches.”

**Introduction**

Q8. Suggest deleting “the psychometrics of” from this sentence: “Regarding empathy in the physician-patient relationship, the psychometrics of the patient’s view of the physician’s…” and changing the word “compliance” to “adherence”

A8. As indicated, the wording has been changed.

Q9. Instead of “cancer patients” use “patients with cancer”

A9. As indicated, the wording has been changed.

Q10. Suggest deleting “the sense of” in this sentence: “there is a lack of empirical data about the sense of empathy in genetic counselors.”

A10. As indicated, the sentence has been changed.

Q11. Change: “. The aim of this pilot study is to reveal the clients’ perceptions of empathy in the genetic counseling context by analyzing simulated sessions.” To ”. The aim of this pilot study was to explore counsellors’ and clients’ perceptions of empathy in the genetic counseling context by analyzing simulated sessions.”

[amemdment: having read the whole manuscript, it seems to be about THE PROCESS through which empathy is experienced in genetic counseling, rather than just empathy in general - suggest rewording the above accordingly]

A11. Thank you for your valuable feedback. This sentence has been corrected to “ The aim of this pilot study was to explore the process through which counselors and clients experienced empathy in the genetic counseling context by analyzing simulated consultations.”

Q12. Change: “However, IPR was difficult to adopt for clients in clinical settings for the feasibility of this study that was conducted to fulfill a degree requirement. Therefore, our survey targeted simulated genetic counseling session” to “Due to the difficulties associated with using IPR for clients in clinical settings, our survey targeted simulated genetic counseling session…”

A12. Thank you for your suggestion. As indicated, the sentence has been changed, and the term “consultations” has been used instead of “sessions.”

**Methods**

Q13. More information is required about the methodology chosen. Specifically why was grounded theory chosen ? How was it actually operationalized within a constructivist paradigm? This sentence makes it sounds actually rather like the approach taken was closer to positivist or post positivist than constructivist: “Coding and theorizing procedures were repeated to reach consensus between both author”. Suggest the authors examine https://onlinelibrary.wiley.com/doi/10.1002/jgc4.1644 Further, what was the underlying conceptual framework?

A13. Thank you for your comment. As per your suggestions, the Data analysis section of our Methods has been thoroughly rephrased.

Q14. The following suggests that the authors are alluding to the concept of “saturation”: “Data collection was completed when new categories were not generated after new data addition” - however, the specifics and evidence of having reached saturation is missing.

A14. Thank you for your comment. The concept of “theoretical sufficiency” and its specifics have been added in the Data analysis section.

**Results**

Q15. The data provided are very thin there are very few direct quotes from participants. the value of qualitative approaches is typically within the richness of the data, the depth of understanding of participants perspectives and experiences. In this manuscript, the few quotes that are provided are all within table 4, and are separated into counselor and client quotes. This presentation feels very positivist and does not allow for true appreciation of the way in which the different components of the model interrelate.

The authors indicate that they built a theoretical model - it’s not clear what exactly the model is of (perhaps see the comment above about the process of developing , and there is no overarching description of how all of the components of the model fit together.

A15. Thank you for your valuable comments. The Results and Discussion sections have been thoroughly rephrased.

**Reviewer #2**:

Q1. The abstract is not as clear as the rest of the paper and includes some grammatical errors. Editing the abstract to ensure the meaning is clear will help increase the profile of this work.

A1. Thank you for your comment. The abstract has been revised to clarify meanings and correct any grammatical errors.

Q2. The term simulation can be used to mean a simulated consultation (as in this paper) or a learning experience (as described in a recent genetic counselling paper by Jacobs & McEwen, 2021). Simulation can also be used as a form of assessment. It would be helpful to include a definition of simulation as used by these authors, and to consider whether simulated consultation is a more appropriate term.

A2. Thank you for your comment. We have changed our use of the term “simulation” to “simulated consultation” at all relevant instances in the manuscript.

Q3. The authors discuss the possibility of using simulation to develop empathy in genetic counselling education and training. Including literature where simulation has been used with learning outcomes that include developing effective client-counsellor relationships would further support the discussion. This paper may be of interest.

Jacobs, C. and McEwen, A. (2021). Adapting to the challenges of the global pandemic on genetic counselor education: Evaluating students’ satisfaction with virtual clinical experiences. J Genet Couns. 2021;30:1074–1083 DOI: 10.1002/jgc4.1490

A3. Thank you for your helpful suggestion. We have added the paper (Jacobs & McEwen 2021) in the reference list.
