# Supplementary material for: Clients’ and genetic counselors’ perceptions of empathy in Japan: A pilot study of simulated consultations of genetic counseling
Source: PLoS One. 2023 Jul 19;18(7):e0288881. doi: 10.1371/journal.pone.0288881 (PMC10355444; doi:10.1371/journal.pone.0288881)
Supplement: Attachment — Submitted filename: Response_to_Reviewers2.doc [file pone.0288881.s002.doc]

**Reviewer #1**:

**Abstract**:

Q1. Suggest changing the first two sentences of the abstract to read as follows:

The rapid increase of the availability of genetic testing is driving the acceleration of genetic counseling implementation. Empathy is important in medical encounters in general, and forms a core component of a successful genetic counseling session, but there ilittle empirical data about empathy in genetic counseling.

A1. As recommended, the first two sentences have been revised.

**Methods**

Q2. The authors discuss that they are male and female, and expect that the participants would be female… but its not actually clear in how the language is being used whether the authors are referring to sex or gender. Clarity on this would be useful. Especially as the discussion comments: “There are also cultural and gender differences in communication characteristics [43,44], and our data might only be representative of the perspectives of Japanese women.” Please note that while “women” is a term that refers only to gender and not sex, “female” is typically used to refer to sex but can also be used to refer to gender.

A2. Thank you for your valuable suggestion. As per your recommendation, we have revised the sentence under the section on Researchers to clarify the cisgender identity of the research team members. Further, the phrase “women” has been changed to “females.”

Q3. It would also be useful to make explicit whether counsellor and client IPR interviews were conducted separately or together.

A3. Thank you for your valuable suggestion. As per your recommendation, the fact that counsellor and client IPR interviews were conducted individually has been added.

Q4. How was it decided which sections of the video related to empathy, and who made that decision? This should be included in the manuscript.

A4. As indicated, the sentence has been revised to highlight that each interviewee decided the sections of the video related to empathy.

Q5. The rationale for choosing grounded theory as a methodology is not strong as currently written. Specifically, there are many approaches that can apply the constant comparative method ( described in lines 157-132), rather not all qualitative methodologies aim to produce a theory of a process - this seems to be the most compelling reason to choose this methodology for this work. Suggest rewording accordingly.

A5. As indicated, the rationale for choosing the method has been revised to highlight the suitability of grounded theory for the purpose of producing a theory.

Q6. Please check through the manuscript and ensure that themes are not referred to as “emerging” (e.g. p31)n- as this term suggests that they are objectively present, which runs counter to the premise of constructivism.

A6. As per your recommendation, the word “emerged” has been changed to “were generated.”

**Discussion**

Q7. Having participated as a counsellor in a simulated genetic counseling session, I wonder whether there is any chance that the fact that it is simulated might interfere with or influence the ways in which empathy manifests in a session. This might warrant mention in the discussion (if there is any literature about this) or limitations.

A7. Thank you for your valuable comments. We searched the literature for evidence regarding whether a simulated situation influenced the ways in which empathy manifests; however, no clear evidence was found. We have mentioned the possibility of such an influence in the Discussion and have added the following reference regarding practitioners’ behaviors in simulations:

[43] Boss RD, Donohue PK, Roter DL, Larson SM, Arnold RM. “This is a decision you have to make”: using simulation to study prenatal counseling. Simul Healthc. 2012;7: 207–212. doi: [10.1097/SIH.0b013e318256666a](https://doi.org/10.1097/SIH.0b013e318256666a)
